# Development & Validation of Urdu Receptive Language Scale (URLS)

**DOI:** 10.12669/pjms.37.7.3928

**Published:** 2021

**Authors:** Ghazal Awais Butt, Nazia Mumtaz, Ghulam Saqulain

**Affiliations:** 1 Ms. Ghazal Awais Butt, M.S (Speech Language Pathology) Speech Language Pathologist, Department of Speech Language Pathology, Riphah International University, Lahore, Pakistan; 2 Dr. Nazia Mumtaz, PhD (Rehabilitation Sciences) Head of Department, Department of Speech Language Pathology, Faculty of Rehab and Allied Health Sciences, Riphah International University, Lahore, Pakistan; 3 Dr. Ghulam Saqulain, F.C.P.S. (Otorhinolaryngology) Head of Department, Department of Otorhinolaryngology, Capital Hospital PGMI, Islamabad, Pakistan

**Keywords:** Receptive language, Reliability, Tool, Urdu, Validity

## Abstract

**Objectives:**

To develop “Urdu Receptive Language Scale (URLS)” for Urdu speaking Pakistani children of age 0-6 years.

**Methods:**

This exploratory study was done at mainstream schools and day care centers on children with normal language development between the ages of 0-6 years from 1^st^ March 2016 to 31^st^ August 2016, by using convenient sampling technique. Firstly, the items for the questionnaire were constructed from four sources: literature review, experts, parents and direct observation of 384 Children of same age. Secondly the constructed test items were sent to the field experts (SLP’s) for the purpose of improvement. Thirdly, after incorporation of suggestions, the improved items were securitized by Urdu experts and finalized. In the next step, these items were tested for Relevance, Ambiguity, Clarity and Simplicity from field experts. The developed scale was then analyzed for reliability and validity by SPSS Version-18.

**Results:**

Study resulted in a 59 items Urdu Receptive Language Scale with each age range having different test items distribution. The mean of the relevance, clarity, simplicity, and ambiguity of test items was 3.89. The Item content validity index value was one for each of the 59 items. The content validity index for the entire scale was also one. The Cronbach’s alpha was 0.948, which indicates a high level of internal consistency.

**Conclusion:**

The developed 59 Item Urdu Receptive Language Scale is reliable and valid tool for language assessment of Urdu speaking Pakistani children of 0-6 years age.

## INTRODUCTION

Voice, speech and language are tools essential for human communication. Voice is produced by the vibration of vocal cords, transformed into Speech, which is the production of recognizable sounds by the articulators. Language is the act of speech with intuitions of an individual transformed into words of a language,^1^ hence it is a system of conventional signs and symbols that people of particular region or community use to express ideas and feelings. Language contains socially shared codes and rules to communicate in a meaningful way and it can be expressed in verbal, non-verbal forms, both being important.^2^ Language can be classified as receptive and expressive language.^3^ Receptive language signifies comprehension, pertaining to critical elements of understanding of commands, stories and directions. While, expressive language refers to expression of language by employing appropriate forms of the language like nouns, adjectives, verbs and prepositions.

English language is a fixed word order dialect and takes after the Subject-Verb-Object structure, while Urdu language is a free word-order dialect and permits numerous conceivable word orderings yet the most well-known sentence structure utilized by the local speakers is Subject-Object-Verb (SOV). Both dialects contrast in morphological and syntactic elements. English has a moderately basic inflectional framework: just nouns, verb and adjectives can be arched, and the quantity of conceivable inflectional joins is entirely little while Urdu is exceedingly inflectional and rich in morphology. In Urdu, the verbs are bent by gender, number and individuals of the head noun, noun phrases are set apart for gender orientation, number and case and adjectives bend as indicated by the gender and number of the head noun. Likewise, rather than English prepositions, Urdu verbs and nouns are trailed by postpositions.^4^

The speech and language skills depend on rich exposure of these skills. The first three years of life are critical for acquiring language skills and failure to get ample exposure during this vital period results in delayed learning.^5^ In the process of language development, the milestones of a child act as a checkpoint for an average child that he/she should attain at a particular age. This is also useful for professionals, teachers and parents to understand the normal language development process and identify delays if any.

Speech-language pathologists (SLP) need to perform formal and informal assessments that provide information of a child’s understanding and/or use of progressively complex aspects of language in order to identify and support the receptive and expressive language learning needs of diverse children during their early learning experiences. However, evidence regarding types of methods and tools in practice are lacking. A number of standardized tools are available in English language for assessment including New Reynell Developmental Language Scales (NRDLS)^4^, which is a standardized assessment tool for language comprehension and production for children 2-7 years of age with items representing language acquisition and impairment. Another commonly used scale is the Clinical Evaluation of Language Fundamentals -Preschool-2 (CELF-Preschool-2), a standardized tool used for the assessment of language skills in preschool children and assess receptive and expressive language skills.^5^ Also Preschool Language Scale-3 (PLS 3) is a standardized tool used for the assessment of receptive and expressive language skills in children, aged range two weeks to six years and consists of two sub scales and three optional supplemental measures. The use of auditory comprehension subscale is to assess the understanding of receptive language skills in the areas of attention, semantics, morphology, syntax and integrative thinking skills. The expressive communication subscale is used to evaluate expression of language in the areas of vocal development, social communication, semantics, morphology, syntax and integrative thinking skills. The three optional measures in PLS-3 are Articulation Screener, Language Sample Checklist, and Family Information and Suggestions Form.^6^ Another scale is the British Picture Vocabulary Scale (BPVS), which is used to assess the comprehension of single words in 3 to 16 years old kids.^7^ Presently the only tool available for assessment in Urdu language assesses only spoken vocabulary skills.^8^

Early intervention for the child with language delay is crucial to social, affective and intellectual development and better adjustment. However, intervention cannot be initiated without a valid and reliable assessment of the child’s current level of functioning. Currently there is no standardized tool available for Urdu language assessment and informal screening forms are used to assess these parameters.

Hence, the current study was designed with the objective to develop an Urdu Receptive Language Scale (URLS), for Urdu speaking Pakistani children between the ages of 0-6 years. Since majority of the population of the country is Urdu speaking, and language assessment is essential in most speech and language disorders^9^ this study is important because with URLS improved assessments of children with language impairments would be possible, leading to early intervention paving way for better management. Also this study might have implications for future research in the field of SLP, the current URLS being the only available tool for Pakistani Urdu speaking population.

## METHODS

Current Exploratory research was conducted over a period of six months from 1st March, 2016 to 31^st^ August, 2016. Sample included 5 expert Urdu linguists & five expert Speech and language pathologists, with minimum Master’s degree; while for direct observation, a sample of n=384 children and their parents were recruited from mainstream schools and day care centers, using convenience sampling technique. Sample size was calculated using Raosoft online calculator with a confidence level of 95%, 5% margin of error and a population size of 50,000. The sample included children of both genders with normal language development of age range of 0-6 years, having Urdu language as their native language. Children with speech and language disorders and with any disability, and children whose parents refused to be included in the study were excluded.

Study was conducted following ethical approval of Institutional Research Board of Riphah International University vide (Ref: REC/RCRS/16/3001, Dated 15^th^ February, 2016 and informed consent of the participant experts and parents of children. Confidentiality of the participants was maintained. Data was collected from experts, parents and by direct observation of children. The sample of children N=384 was divided into 12 age groups including 0-6, 7-12, 13-18, 19-24, 25-30, 31-36, 37-42, 43-48, 49-54, 55-60, 61-66 and 67-72 months. The study was divided into two phases: Phase 1 included development of Urdu Receptive Language Scale (URLS) and Phase 2 included reliability and validity testing of the same.

### Phase 1: Development of the URLS Scale

Firstly, the items for the questionnaire were constructed from four sources: literature review, experts, parents and direct observation of n=384 children, Secondly the constructed test items were sent to the experts, Thirdly, after the improvement of questionnaire these items was again sent to Urdu experts and were finalized, Fourthly, items were tested for Relevance, ambiguity, clarity and simplicity from experts. The positive responses from experts were termed as agreed and presented by code X and score- 1, and negative responses were presented by code 0.

### Phase 2: Testing of Reliability and validity of the URLS Scale

After the development of the scale, the reliability and validity of the scale was analyzed.

SPSS 18 was used for data entry and analysis. Descriptive statistics along with Cronbach’s alpha reliability analysis and Content validity index.

## RESULTS

The tryout sample (N=384) with male female ratio of 1:1, with 32(8.33%) children in each of the 12 age groups (0-6, 7-12, 13-18, 19-24, 25-30, 31-36, 37-42, 43-48, 49-54, 55-60, 61-66 and 67-72 months) (Table-I). Experts included 5 SLP’s, and 5 Urdu Language experts including 2 PhD’s as well. Most 8(80%) experts were experienced with more than five years’ experience.

**Table I:** Demographic distribution of sample of tryout subjects (n = 384) & Experts (n = 10).

*Sample Group*	*Variable*	*Category*	*N(%)*
Tryout Subjects (N=384)	Gender	Male	192 (50)
		Female	192 (50)
	Age Group	0-6 months	32 (8.33)
		7-12 months	32 (8.33)
		13-18 months	32 (8.33)
		19-24 months	32 (8.33)
		25-30 months	32 (8.33)
		31-36 months	32 (8.33)
		37-42 months	32 (8.33)
		43-48 months	32 (8.33)
		49-54 months	32 (8.33)
		55-60 months	32 (8.33)
		61-66 months	32 (8.33)
		67-72 months	32 (8.33)
Experts Qualification (N=10)	Speech Language Pathology (n=5)	MS/MPhil	5 (50)
	Urdu Language (n=5)	MS/MPhil	3 (30)
		PhD	2 (20)
Experts Experience (N=10)	SLP	≤ 5 Years	1 (10)
		5-10 Years	4 (40)
	UL	≤ 5 Years	1 (10)
		5-10 Years	2 (20)
		≥10 Years	1 (10)

The Urdu URLS contained a total of 59 test items, each age range having a different test items distribution including 7 items for 7-12 years age group; 6 items for 49-54 years age group; 5 items for 19-24 years and 31-36 years age group each; 4 items for 0-6 years, 13- 18 years, 25-30 years, 37-42 years, 55-60 years, 61-66 years, and 67- 72 years age group each.

**Fig.1:**
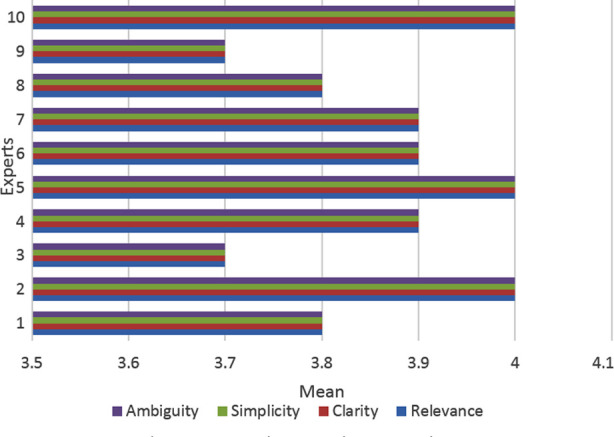
Experts Rating of Content Validation of Urdu Receptive Language Scale.

As regards the content validity of URLS, the Mean±SD for “relevance”, “clarity”, “simplicity” and “ambiguity” was found to be 3.89±0.15 each, with slight variability for individual expert rating of content validity (Fig.1).

As regards Reliability of our 59 item URLS, Cronbach’s alpha value was 0.948, which indicates high level of internal consistency for the scale. For purpose of scoring of the URLS, score below 35 indicated severe problem while that above 53 indicated no apparent problem (Table-II).

**Table II:** Score Interpretation of URLS.

*Total score 59*	*Type of severity*
Above 53	No apparent problem
47-53	Mild
35-47	Moderate
Below 35	Severe

## DISCUSSION

Tools meant for diagnosis developed in a country or region may not be applicable elsewhere.^10^ Also dearth of language assessment tools exists^11^, especially in Urdu language. Though some assessment tools have been developed in Urdu language including test of articulation and phonology in Urdu^12^, however no standardized tool for Urdu language assessment currently available in Pakistan, and use of informal screening forms and self-translated assessment tools being a norm like some other countries.^13^ Also kids having reading disorders not related to attention or movement issues have lower skills for receptive language compared to expressive.^14^ Also to manage specific learning disorders, it is necessary to screen children at initiation of schooling.^15^ Hence, in the present study Urdu Receptive Language Scale (URLS) has been developed to assess the receptive language skills in infants and young children of 0-6 years. Special emphasis has been given to the fact that even in the assessment of preschool children both receptive as well as expressive language is assessed including assessment of more than one language dimension.^16^

The receptive skills’ precursors were evaluated in auditory comprehension domain including tasks focusing on attention in the preschool language scale adopted for children speaking Bangla^17^, with some similarity in the current study, the precursor to receptive skills was evaluated within the receptive language scale with emphasis on tasks that focus on attention, understanding language structures and pragmatics. Receptive and expressive language was represented as consisting of the interrelated components of form, content, and use.^18^ The receptive language scale tasks were used to evaluate receptive language skills in the areas of attention, comprehension development, semantics: vocabulary and concepts, structure: morphology and syntax and integrative thinking skills.^19^ Attention is an essential component since before child learns to grasp language, he must attend to auditory input with resultant improved development of receptive as well as expressive language.^20^ Similarly for the progress of semantics, child learns what words mean, hence growth of receptive vocabulary from few to thousands of words occurs. A child rapidly learns and recognizes the words naming objects like ball, cat; actions like jumping, playing; and understanding these words progresses through learning from immediate neighboring environment and through pictures in books. The morphology and syntax develop along with ability to understand words and sentence structure comprising of few words’ phrases.^21^ According to Cheung EYM, more time is taken for the syntactic acquisition of function words.^22^

Urdu receptive language scale was developed to assess language of children between the ages of 0-6 years. The scale was divided into 12 sections with the difference of six months of age. Each age range includes different number of test items. These items were focusing on attention, listening, command following, understanding questions, morphological structures and making simple conclusions.

In spite of facing difficulties in the scale development process^23^, the scale was developed with the following different steps, including the construction of items by different sources from literature, experts, parents and observation of children. Firstly, test items were constructed and then the language of n=384 children were observed. Data was also collected from parents about their children language development. Then those test items were again discussed with experts. After that these items were tested for relevance, clarity, ambiguity and simplicity. Different English tools like preschool language scale and clinical evaluation of language fundamentals are also constructed following similar steps. There is total 59 receptive language test items that are divided into different age ranges. The results of this study are consistent with the study of Zimmerman IL et al.^6^ study related to development of preschool language scale for infants and children, for receptive and expressive language. The scoring of our Urdu expressive language scale also relates to preschool language scale which is comprised of categories for measuring severity like “no problem”, “mild language problem”, “moderate language problem” and “severe language problem”. The results of this current study are also related to New Reynell Developmental Language Scales (NRDLS) which is a norm referenced test, which assesses the language comprehension and production for children of 2-7 years. The items included in scale reflect the language acquisition and impairment.^4^

Current study is instrumental in adding a reliable Urdu receptive language scale (URLS) to the literature with a good reliability having a Cronbach’s alpha value of 0.948. This is the only tool available for receptive language assessment in Urdu language. This is clinically very significant since, Urdu being national language is well understood all over the country and hence, this tool will help speech language pathologists in early and better assessment of children with language disorders and hence make early management possible. It will also be useful for research in the field.

### Limitations & Suggestions

Though the current study has reported high reliability of the developed tool, it is limited to one city only. Hence, further research is required in rural areas predominantly using local languages to increase generalizability. Furthermore, clinical control trials involving larger samples from different provinces speaking different local languages, can add clinical knowledge as well as guide towards appropriate management strategies.

## CONCLUSIONS

The developed Urdu Receptive Language Scale (URLS) is a reliable and valid tool for language assessment of Urdu speaking Pakistani children 0-6 years of age.

### Authors Contribution:

**GAB, NM** did the manuscript writing.

**NM** conceived, designed and did the editing of manuscript.

**GS** did the literature review and responsible for integrity of the research.
